# Multidrug-Resistant and Clinically Relevant Gram-Negative Bacteria Are Present in German Surface Waters

**DOI:** 10.3389/fmicb.2019.02779

**Published:** 2019-11-29

**Authors:** Linda Falgenhauer, Oliver Schwengers, Judith Schmiedel, Christian Baars, Oda Lambrecht, Stefanie Heß, Thomas U. Berendonk, Jane Falgenhauer, Trinad Chakraborty, Can Imirzalioglu

**Affiliations:** ^1^Institute of Medical Microbiology, Justus Liebig University Giessen, Giessen, Germany; ^2^German Center for Infection Research (DZIF), Partner Site Giessen-Marburg-Langen, Justus Liebig University Giessen, Giessen, Germany; ^3^Bioinformatics & Systems Biology, Justus Liebig University Giessen, Giessen, Germany; ^4^Norddeutscher Rundfunk, Hamburg, Germany; ^5^Institute of Hydrobiology, Technical University of Dresden, Dresden, Germany; ^6^Department of Microbiology, University of Helsinki, Helsinki, Finland

**Keywords:** ESBL, surface water, WGS (whole genome sequencing), MCR-1, clinical isolate

## Abstract

Water is considered to play a role in the dissemination of antibiotic-resistant Gram-negative bacteria including those encoding Extended-spectrum beta-lactamases (ESBL) and carbapenemases. To investigate the role of water for their spread in more detail, we characterized ESBL/Carbapenemase-producing bacteria from surface water and sediment samples using phenotypic and genotypic approaches. ESBL/Carbapenemase-producing isolates were obtained from water/sediment samples. Species and antibiotic resistance were determined. A subset of these isolates (*n* = 33) was whole-genome-sequenced and analyzed for the presence of antibiotic resistance genes and virulence determinants. Their relatedness to isolates associated with human infections was investigated using multilocus sequence type and cgMLST-based analysis. Eighty-nine percent of the isolates comprised of clinically relevant species. Fifty-eight percent exhibited a multidrug-resistance phenotype. Two isolates harbored the mobile colistin resistance gene *mcr-1*. One carbapenemase-producing isolate identified as *Enterobacter kobei* harbored *bla*_VIM–__1_. Two *Escherichia coli* isolates had sequence types (ST) associated with human infections (ST131 and ST1485) and a *Klebsiella pneumoniae* isolate was classified as hypervirulent. A multidrug-resistant (MDR) *Pseudomonas aeruginosa* isolate encoding known virulence genes associated with severe lung infections in cystic fibrosis patients was also detected. The presence of MDR and clinically relevant isolates in recreational and surface water underlines the role of aquatic environments as both reservoirs and hot spots for MDR bacteria. Future assessment of water quality should include the examination of the multidrug resistance of clinically relevant bacterial species and thus provide an important link regarding the spread of MDR bacteria in a One Health context.

## Introduction

The recent emergence of antibiotic-resistant Gram-negative bacteria, in particular those encoding extended-spectrum beta-lactamases (ESBL) and/or carbapenemases, poses a significant threat for human and animal health. Nowadays, they account for a large proportion of the global pandemic spread of antimicrobial resistance (World Health Organization [WHO], 2017). ESBL/Carbapenemase-producers have been isolated from various sources including both animal- and human- populations, as well as from environmental sources including water bodies (Woodford et al., 2014; Grundmann et al., 2017; Hölzel et al., 2018; Huijbers et al., 2015; Madec et al., 2017; Muraleedharan et al., 2019).

An understanding of the causes for the emergence of ESBL/Carbapenemase-producing isolates and probable pathways of their spread would be aided by a deep genetic characterization of isolates from the different sources to detect those clones/antibiotic resistance determinants that are drivers of antibiotic resistance spread. Isolates from humans, animals and food have been studied for a long time, and whole-genome-based approaches have identified identical clones circulating among these compartments (Falgenhauer et al., 2016a, b). Far less is known for aquatic environments, where analyses are mostly based on the phenotypic analysis of antibiotic susceptibility or quantification of resistance genes (Hembach et al., 2017; Müller et al., 2018). Whole-genome-based studies have been performed on isolates from raw and treated wastewater (Gomi et al., 2018; Mahfouz et al., 2018), but are scarce for other water bodies, in particular recreational water.

To gain more insight into this topic, an investigation was performed in collaboration with investigative journalists in Northern Germany (Baars and Lambrecht, 2018). This resulted in the detection of antibiotic-resistant Gram-negative isolates in surface water bodies, partly used for recreational purposes that depicted multilocus sequence types found previously in hospital settings and are representative of relevant human pathogens. Here we report on these findings.

## Materials and Methods

### Sampling and Detection of ESBL-Producing Isolates

Water (*n* = 12) and sediment (*n* = 10) samples were taken from different water bodies located in northern Germany (Supplementary Figure S1 and Supplementary Table S1) in autumn 2017. Sampling sites were selected for their relevance to resistance spread (e.g., neighborhood to human clinics, recreational water bodies or food processing plants). All samples were processed within 24 h. For detection of viable ESBL/Carbapenemase-producing bacteria in the water samples, four different volumes (exact volumes differed from sample to sample depending on the expected “contamination” level) were filtered (two technical replicates per sample; pore size 0.2 μm, cellulose acetate filters, Sartorius; Göttingen, Germany). The filters were put onto CHROMagar ESBL^®^ plates (MAST Diagnostica, Reinfeld, Germany) and incubated for 18 ± 2 h at 42°C. Sediment samples were supplemented with sodium pyrophosphate solution (20 mL 0.1% solution/100 g wet sediment) and shaken (460 rpm, room temperature, 30 min). Two-hundred microliters of the supernatant were subsequently plated on CHROMagar ESBL^®^ plates and incubated as described above.

### Species Determination and Antibiotic Susceptibility Testing

A subset of bacterial isolates (*n* = 134) growing on selective agar were characterized by VITEK^®^ MS (BioMérieux, Nüertingen, Germany) for the determination of species. Antibiotic susceptibility testing was performed using the VITEK^®^2 system following to the manufacturer’s instructions (BioMérieux, Nüertingen, Germany). Minimal inhibitory concentrations were interpreted according to EUCAST, 2017 and CLSI, 2016 guidelines (internal VITEK^®^2 adjustment) The multidrug-resistance (MDR) status was evaluated for seven clinically relevant species (*Acinetobacter baumannii, Citrobacter freundii, Enterobacter cloacae complex, Escherichia coli, Klebsiella oxytoca, Klebsiella pneumoniae*, and *Pseudomonas aeruginosa*) using the classification system of Magiorakos et al. (2012), with resistance to ≥3 classes of antibiotics defining the MDR status.

### Whole Genome Sequencing and Genome-Based Analysis

Thirty-three isolates out of the 134 phenotypically characterized isolates (Table 1) were subjected to whole-genome sequencing and subsequent analysis. These isolates included seven different species (*A. baumannii*, *Enterobacter cloacae* complex, *E. coli, K. oxytoca, K. pneumoniae, P. aeruginosa*, and *Pseudomonas resinovorans*). If more than one isolate of an identical species was present at one sampling site, isolates representative of different antibiotic resistance phenotypes were chosen.

**TABLE 1:** Characteristics of the sequenced isolates.

**Isolate**	**Species**	**MLST**	**Beta-lactamase**	**Colistin**	**Tetracycline**	**Phenicol**	**Aminoglycoside**	**Fosfomycin**	**Macrolide**	**Sulfonamide**	**Fluoroquinolone**	**Trimethoprim**	**Lincosamide**
1w-12	*A. baumannii*	203^%^	*bla*_ADC–__25_, *bla*_OXA–__78_										
2w-4	*A. baumannii*	155^%^	*bla*_ADC–__25_, *bla*_OXA–__51_										
3w-14	*A. baumannii*	1017^%^	*bla*_ADC–__25_, *bla*_OXA–__203_										
6s-1	*A. baumannii*	1321^%^	*bla*_ADC–__25_, *bla*_OXA–__51_										
6w-10	*A. baumannii*	1322^%^	*bla*_ADC–__25_, *bla*_OXA–__69_										
7w-12	*A. baumannii*	647^%^	*bla*_ADC–__25_, *bla*_OXA–__208_										
8s-2	*A. baumannii*	1112^%^	*bla*_ADC–__25_, *bla*_OXA–__88_										
8s-4	*A. baumannii*	1112^%^	*bla*_ADC–__25_, *bla*_OXA–__88_										
8w-16	*A. baumannii*	690^%^	*bla*_ADC–__25_, *bla*_OXA–__71_										
8w-19	*A. baumannii*	1323^%^	*bla*_ADC–__25_, *bla*_OXA–__93_										
10w-8	*A. baumannii*	1324^%^	*bla*_ADC–__25_, *bla*_OXA–__208_										
3w-16	*A. pittii*	249^%^	*bla*_ADC–__25_, *bla*_OXA–__421_										
12w-5	*Enterobacter kobei*	910	*bla*_VIM–__1_				*aadA1, aadB, aacA4*	*fosA*		*sul1*	*aac(6*′*)Ib-cr*		
1w-4	*E. coli*	2064^$^	*bla*_CTX–M–__1_										
1w-5	*E. coli*	648^$^	*bla*_CTX–M–__14_										
2w-3	*E. coli*	167^$^	*bla*_CTX–M–__1_		*tet(Y)*	*floR*	*aac(3)-IVa, aadA1, aph(4)-Ia, strA, strB*		*mph(A)*			*dfrA1*	
3w-1	*E. coli*	101^$^	*bla*_CTX–M–__1_				*aadA5*			*sul2*		*dfrA17*	
3w-4	*E. coli*	10^$^	*bla*_CTX–M–__15_, *bla*_OXA–__1_, *bla*_*TEM–*__1_	*mcr-1*	*tet(B)*	*catB3*	*aadA17*				*aac(6*′*)Ib-cr*		*lnu(F)*
6w-2	*E. coli*	9417^$^	*bla*_CTX–M–__1_				*aadA5*			*sul2*		*dfrA17*	
7w-2	*E. coli*	542^$^	*bla*_CTX–M–__1_				*aadA1*						
7w-4	*E. coli*	69^$^	*bla*_CTX–M–__15_		*tet(B)*				*mph(A)*			*dfrA14*	
8w-1	*E. coli*	1485^$^	*bla*_CTX–M–__15_, *bla*_*TEM–*__1_								*qnrS1*		
8w-3	*E. coli*	155^$^	*bla*_CTX–M–__1_, *bla*_*TEM–*__1_	*mcr-1*	*tet(A)*		*aac(3)-IIa, aadA1, aph(3*′*)-Ic, strA, strB*			*sul2*		*dfrA1*	
9w-1	*E. coli*	131^$^	*bla*_CTX–M–__1_						*mph(A)*				
10w-3	*E. coli*	10^$^	*bla*_CTX–M–__15_, *bla*_OXA–__1_		*tet(B)*	*catB3*					*aac(6*′*)Ib-cr*		
1w-9	*K. oxytoca*	241	*bla*_*OXY–*__2__–__2_										
3w-10	*K. oxytoca*	34	*bla*_*OXY–*__2__–__8_				*strA, strB*		*mph(A)*	*sul2*			
1w-10	*K. pneumoniae*	3681	*bla*_CTX–M–__15_, *bla*_OXA–__1_, *bla*_SHV–__28_		*tet(D)*	*catB3*	*strA, strB*	*fosA*		*sul2*	*oqxA, oqxB, aac(6*′*)Ib-cr*	*dfrA14*	
3w-9	*K. pneumoniae*	307	*bla*_CTX–M–__15_, *bla*_OXA–__1_, *bla*_SHV–__28_, *bla*_*TEM–*__1_			*catB3*	*strA, strB*	*fosA*		*sul2*	*oqxA, oqxB, aac(6*′*)Ib-cr*	*dfrA14*	
7w-11	*K. pneumoniae*	268	*bla*_CTX–M–__15_, *bla*_SHV–__11_					*fosA*	*ere(A)*	*sul1*	*oqxA, oqxB*	*dfrA5*	
8w-11	*K. pneumoniae*	2155	*bla*_SHV–__2_		*tet(D)*		*aac(3)-IId, strA, strB*	*fosA*		*sul2*	*oqxA, oqxB, qnrS1*	*dfrA14*	
9w-9	*P. aeruginosa*	3304	*bla*_OXA–__50_, *bla*_PAO_			*catB7*	*aph(3*′*)-IIb*	*fosA*					
10w-9	*P. resinovorans*	ND	*bla*_POM–__1_										

*ND: not determined due to the lack of a known MLST scheme. ^%^ MLST determined using Pasteur Scheme (Diancourt et al., 2010), ^$^ MLST determined using Achtman scheme (Wirth et al., 2006). The first number in the name of the isolates depicts, from which sampling site they were isolates (as described in Supplementary Table S1); s, source soil, w, source water.*

DNA was isolated from overnight cultures using the PureLink^®^ Pro 96 Genomic DNA Purification Kit (Thermo-Fisher Scientific, Dreieich, Germany). For short-read sequencing, an Illumina Nextera XT library was prepared and sequenced on a NextSeq 500 device (Illumina, Eindhoven, Netherlands) using the NextSeq 500/550 Mid Output v2 kit (2 × 150 nt read length). Sequence data analysis (coverage, mean read length, quality clipping, assembly, determination of multilocus sequence types and virulence genes using the database of VFDB (Chen et al., 2016) was performed using the in-house pipeline ASA^3^P (Schwengers et al., 2019). A mean coverage of 103× and a mean read length of 130 nt was achieved. Quality controlled raw reads were assembled using Spades v.3.10.1 (Bankevich et al., 2012) implemented in the ASA^3^P pipeline, with a mean N50 value of 103,031 nt. The sequencing data was deposited in the ENA database under the accession number PRJEB29745.

Antibiotic resistance genes and plasmid incompatibility groups were analyzed using the bacterial analysis pipeline at the Center for Genomic Epidemiology (Thomsen et al., 2016). *E. coli fimH*-types were determined using the Fimtyper tool (Roer et al., 2017). Virulence genes were additionally analyzed using the bacterial analysis pipeline at the Center for Genomic Epidemiology (Thomsen et al., 2016) and BIGSDb (scheme “virulence genes”) for *K. pneumoniae* isolates^1^.

Assignment of the *Enterobacter* species was performed using the genome sequence of the type strains referred in Supplementary Table S2 and the online GGDC tool (Meier-Kolthoff et al., 2013) applying a threshold of 70% sequence similarity for taxonomic identification.

The determination of the source of different multilocus sequence types (ST) was performed using EnteroBase for *E. coli* (Alikhan et al., 2018) and PubMLST database for *Acinetobacter baumannii* isolates^2^ [Pasteur scheme (Diancourt et al., 2010)].

For a detailed analysis of the source, BacWGSTdb (Ruan and Feng, 2016) was used to determine the closest relative with the function “Single genome analysis” and a core genome MLST (cgMLST) allele threshold based on 200 alleles. The analysis was performed for *E. coli*, *K. pneumoniae*, and *A. baumannii* isolates (*n* = 27), because the BacWGSTdb included schemes for these species.

## Results and Discussion

The role of aquatic environments and wastewaters for the dissemination of antibiotic-resistant Gram-negative bacteria is an increasing source of concern worldwide and has not yet been investigated in detail so far (Berendonk et al., 2015). The presence of both contaminated wastewater and other anthropogenic selection pressures on aquatic environments are likely to create hotspots and reservoirs for the selection of antibiotic-resistant bacteria and the transfer of antibiotic resistance genes. Despite these facts, most water samples are routinely monitored only for the concentration of fecal indicators (*E. coli* and *Enterococcus* sp.; EU Directive 2006/7/EC). It is not mandatory for authorities to store or characterize the detected isolates further. Consequently, no systematic information on the presence of antibiotic resistance genes or the clinical relevance of the isolates is collected.

Several studies have been performed to estimate the role of surface waters for the spread of antibiotic resistance. qPCR-based studies are still the standard procedure in many countries, including Germany (Hembach et al., 2017). The disadvantage of these studies is that they do not allow the direct association of antibiotic resistance genes to a given bacterium. Studies implementing the analysis of viable antibiotic-resistant bacteria for antibiotic resistance phenotype and antibiotic resistance genes do not allow to determine the relationship of the bacteria in water to bacteria from other sources. This is only possible using genome-based studies that are still an exception (Jorgensen et al., 2017). The only genome-based study of water in Germany employs the analysis of wastewater (Mahfouz et al., 2018).

In our study, we combined phenotypic and genotypic analyses of antibiotic-resistant bacteria isolated from water and sediment samples to determine their relationship to bacteria from other sources and to determine their possible role on human health.

### Clinically-Relevant Multidrug-Resistant Gram-Negative Bacteria Are Present in Recreational Water Samples

In 10 out of 12 water samples, ESBL/Carbapenemase-producing Gram-negative bacteria were detected by a culture-based approach, with concentrations varying between 2.7 and 8.1 × 10^3^ colony forming units per 100 mL. In 20% of the analyzed sediment samples, viable ESBL/Carbapenemase-producers were detected (Supplementary Table S3). The lack of positive sediment samples should not be the result of the method used, as it has been shown in an earlier study that the number of cells derived by this method was not increased by ultrasonic treatment (Heß et al., 2018).

**FIGURE 1:**
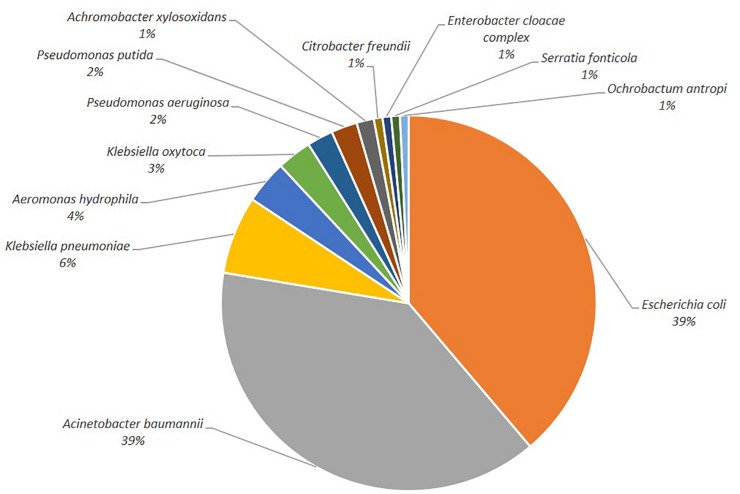
Depiction of the bacterial species distribution.

One hundred-thirty-four isolates growing on selective agar plates were selected for species determination and antibiotic susceptibility testing. The most common species detected were *E. coli* and members of the *A. baumannii* complex (Figure 1, 39%, respectively), followed by *K. pneumoniae* (6%). Other clinically relevant species (*C. freundii, E. cloacae, P. aeruginosa*) were less abundant.

**FIGURE 2:**
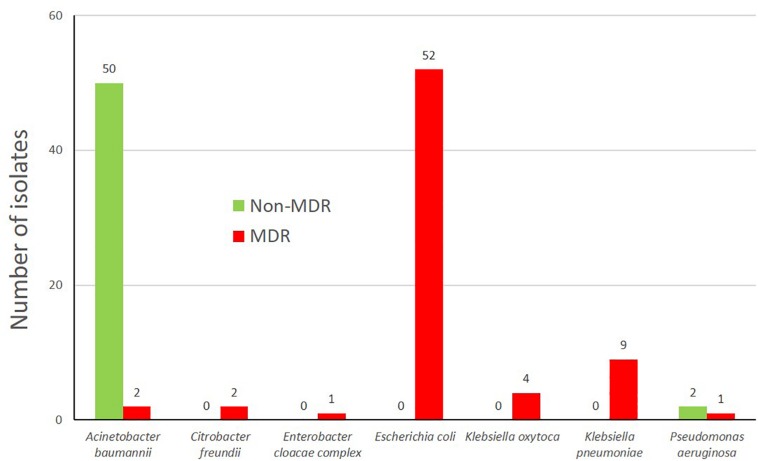
Multidrug status of the bacterial isolates (grown on selective growth medium) according to the classification of Magiorakos et al. (2012).

The multidrug-resistance (MDR) status of seven clinically relevant species (Figure 2) of the isolates was evaluated and varied between 3.8% of a respective species (*A. baumannii* complex) to 100% (*C. freundii, E. cloacae, E. coli, K. oxytoca*, and *K. pneumoniae*, Figure 2). The low number of MDR *A. baumannii* is concordant to data from Austria (Kittinger et al., 2017) where only 4.4% of the *A. baumannii* isolates were MDR. These MDR-isolates were mostly third generation cephalosporin resistant, but also Carbapenem non-susceptible isolates were present within the characterized isolates (*n* = 7) including two isolates of *P. aeruginosa* (9w-9, 10w-5), *A. baumannii* complex (6w-8, 8s-4), *K. oxytoca* (11w-2, 12w-10) and one *E. cloacae* complex isolate (12w-5) (Supplementary Table S4).

### Water Isolates Harbor Antibiotic Resistance Genes Toward Eleven Antibiotic Classes

Genome-based analysis revealed that genes encoding resistance to eleven different antibiotic classes were present in the sequenced isolates (Table 1). All sequenced isolates harbored at least one beta-lactamase gene, accompanied by different phenotypes. The *P. aeruginosa* isolate 9w-9 encoded the *bla*_OXA–__50_ gene (carbapenemase) and *bla*_PAO_ (AmpC gene) beta-lactamase genes, while the *P. resinovorans* isolate (10w-9) harbored the beta-lactamase gene *bla*_POM–__1_. The *A. baumannii* isolates (*n* = 11) harbored the chromosomally-encoded AmpC gene *bla*_ADC__–__25_ in combination with a *bla*_OXA_-like beta-lactamase conferring phenotypic ertapenem resistance (Table 1 and Supplementary Table S4). The *Enterobacter kobei* isolate (12w-5) harbored the carbapenemase gene *bla*_VIM__–__1_.

The most common ESBL genes detected (Table 1) were *bla*_CTX–M–__1_ (*n* = 7) and *bla*_CTX–M–__15_ (*n* = 7). The ESBL gene *bla*_CTX–M–__1_ was found exclusively in *E. coli* isolates, whereas the *bla*_CTX–M–__15_ gene was also detected in *K. pneumoniae*. Less frequent ESBL genes were *bla*_SHV–__28_ (*n* = 2), *bla*_CTX–M–__14_ (*n* = 1) and *bla*_SHV–__2_ (*n* = 1). The ESBL gene *bla*_SHV–__28_ was found only in *K. pneumoniae* and always in combination with *bla*_CTX–M–__15_. The ESBL genes are similar to those found in other studies (Jorgensen et al., 2017), but the distribution was different, as the *bla*_CTX–M–__1_ gene was detected more frequently (*n* = 7) than *bla*_CTX–M–__15_ (*n* = 4). The prevalence of these genes also reflect the situation found in hospital settings in Germany, together with the very low incidence of acquired carbapenemase genes.

With the exception of *A. baumannii*, isolates harbored additional antibiotic resistance genes conferring resistance to ten antibiotic classes (Table 1). The most commonly found resistance genes were those encoding aminoglycoside resistance (12/33, e.g., *strA/strB*, *aadA1*) followed by genes conferring resistance against sulfonamides (*n* = 9, *sul1*, *sul2*), trimethoprim (*n* = 9, *dfrA1*, *dfrA14*, *dfrA17*) and Fluoroquinolones (*n* = 8, *qnrS1*, *oqxA*, *oqxB*, *aac*(*6*′)*Ib-cr*).

Most strikingly, the mobile colistin-resistance gene *mcr-1* was detected in 2/33 isolates, all being *E. coli* isolates. In the isolate 3w-4, the *mcr-1* gene was located on an IncX4 plasmid, while 8w-3 it was located on an IncHI2 plasmid. The isolates harboring *mcr-1* differed from those found in Switzerland (Zurfuh et al., 2016) both by multilocus sequence type and the encoded ESBL gene. The *mcr-1*-encoding isolates were only found in the neighborhood of a meat processing plant (sampling site 3) and a slaughterhouse (sampling site 8), concordant with the finding that *mcr-1* is more prevalent in livestock than in humans (Schwarz and Johnson, 2016).

### Detection of Plasmid Incompatibility Groups Present in the Isolates

The Enterobacteriaceae isolates harbored a number of different plasmid incompatibility groups (Supplementary Table S5 and Supplementary Figure S2). Col-type plasmids were the most frequent plasmids, followed by IncI1 and IncF-type plasmids. IncI1 and IncF plasmids are frequently encoding ESBL genes (García-Fernández et al., 2008; Villa et al., 2010).

### Detection of Virulence Determinants and Phylogeny of the Isolates

The sequenced isolates were subjected to detailed analysis to determine a clinical relevance or possible transmission from/to hospital settings. *K. pneumoniae* isolates belonged to four different multilocus sequence types (Table 1), of which two (ST268 and ST307) have previously been described in human clinical specimens (Zhang et al., 2016; Wyres et al., 2019). The present isolates were isolated at water treatment plants, but also in the neighborhood of a slaughterhouse indicating a possible spread of these into the animal population. ST307 *K. pneumoniae* isolates have emerged worldwide only recently and are supposed to have a high transmission potential, so they are able to quickly move between different countries and even continents (Wyres et al., 2019). They are almost exclusively associated with the carriage of the ESBL gene *bla*_CTX–M–__15_. The *K. pneumoniae* isolate 7w-11 (ST268) harbored determinants discriminatory for hypervirulent *Klebsiella* isolates of the sequence type ST268 (pLVPK plasmid, Integrative conjugative element, Supplementary Figure S3; Zhang et al., 2016). Hypervirulent *K. pneumoniae* are associated with severe infections in younger and healthier persons than other *Klebsiella* (Lee et al., 2017).

For the *E. coli* isolates, eleven different multilocus sequence types were detected (Table 1) of which four (ST10, ST131, ST155, and ST1485) are associated with human clinical infections (Pietsch et al., 2015; Skurnik et al., 2016; Gilrane et al., 2017; Schaufler et al., 2019). For the isolate 9w-1 with the sequence type ST131, *fimH*-typing was performed in order to investigate its relationship among the ST131 lineages. It harbors the *fimH*22 allele, therefore placing it into the ST131-H22 lineage which is associated with both human clinical and animal/environmental isolates (Liu et al., 2018).

Other STs have been previously isolated from animal sources [ST167, ST542 (Maamar et al., 2016; Irrgang et al., 2017)]. The sequence types ST10 (*n* = 2), ST101 and ST648 have been isolated from different sources (humans, animals -wild animals and livestock- and food) (Pietsch et al., 2015; Michael et al., 2017; Irrgang et al., 2018; Schaufler et al., 2019)^3^. ST155 is mainly found in association with livestock and only recently emerged in humans (Skurnik et al., 2016). These results indicate that water samples harbor isolates from different sources. They may even be able to spread throughout different compartments as their relatives. Five of the above mentioned STs have already been detected in recreational water and/or wastewater samples (ST101, ST10, ST69, ST155, and ST131) in Norway (Jorgensen et al., 2017).

All *E. coli* isolates harbored between two and nine virulence determinants, mostly involved in adherence and iron acquisition/transport (Supplementary Table S6). The three most common virulence determinants were *fdeC* (adherence, *n* = 11), enterobactin (iron acquisition/transport, *n* = 10) and the ECP operon (adherence, *n* = 10). According to the virulence gene content, the pathotypes were determined for 7/12 *E. coli* isolates following the schemes presented by Kaper et al. (2004) and Johnson and Russo (2005). All of these were classified as extra-intestinal pathogenic *E. coli* (ExPEC). Within these, two ExPEC subtypes could be determined, including neonatal meningitis-causing *E. coli* (NMEC, 2/12) and uropathogenic *E. coli* (UPEC, 1/12), respectively. In contrast to the study of Jorgensen et al. (2017) enteropathogenic *E. coli* (EPEC) were not detected.

The *P. aeruginosa* isolate 9w-9 was classified as a multidrug-resistant (MDR) isolate. It was of the new ST3304 (one-allele variant of ST274). *P. aeruginosa* ST274 isolates are of high clinical relevance, as they are known to be associated with severe lung infections in cystic fibrosis patients (Fernández-Olmos et al., 2013). The 9w-9 isolate was found in close proximity to a senior residence, which is concordant with its predominant association with immune-compromised patients. The virulence genes found in this *P. aeruginosa* isolate were previously described disease-associated genes (Gellatly and Hancock, 2013) for the production of flagella, pili, alginate, pyoverdine, pyocyanin, and the effector proteins ExoS, ExoT, ExoY. Flagella and pili enable adherence to host cells and movement, while the production of alginate is crucial for biofilm formation. Through the use of pyoverdine and pyocyanin, these bacteria can grow in iron-depleted environments, as e.g., blood, and can cause oxidative stress to the host. The type III secretion system is required for the successful excretion of the virulence effector proteins ExoS, ExoT and ExoY that cause host cell death. The presence of all these virulence determinants qualified this particular isolate as a successful pathogen indicating that water may also be a source for these.

The *A. baumannii* isolates are representative members of ten different sequence types, of which all but one (ST1112) were present only once among the isolates. Five STs have been found in a specific source (Supplementary Table S7). The *A. baumannii* isolates of the sequence types ST690 and ST1017 have been isolated previously from animals only, while ST155 has been isolated predominantly from animals, but also from humans. ST203 was detected in human isolates and ST647 in environmental isolates. These results indicate that there is a transmission of human and animal *A. baumannii* isolates to the water bodies. The *A. baumannii* isolates did not harbor any known virulence genes.

The *E. kobei* isolate belonging to the *E. cloacae* complex harbored a *bla*_VIM–__1_ carbapenemase and had the sequence type ST910. *E. cloacae* complex isolates are nosocomial pathogens often associated with high resistance and also virulence, especially in immunocompromised patients (Mezzatesta et al., 2012).

**FIGURE 3:**
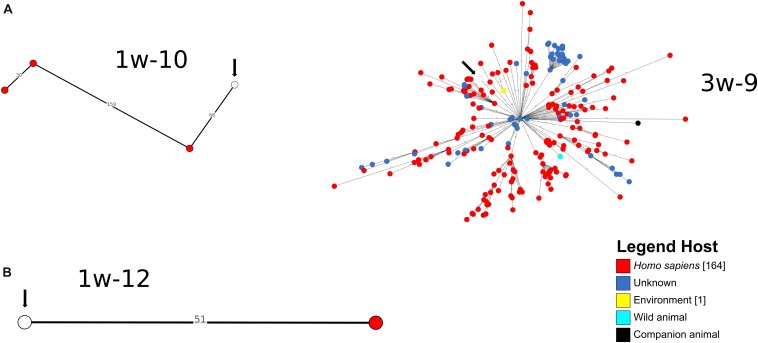
Depiction of cgMLST-based search for closest relatives of sequenced *K. pneumoniae* **(A)** and *A. baumannii* **(B)** isolates. The search for closest relatives was performed using BacWGSTdb (Ruan and Feng, 2016). Different hosts are marked with different colors of the isolates. The water isolate from this study is indicated with a white circle and an arrow.

**FIGURE 4:**
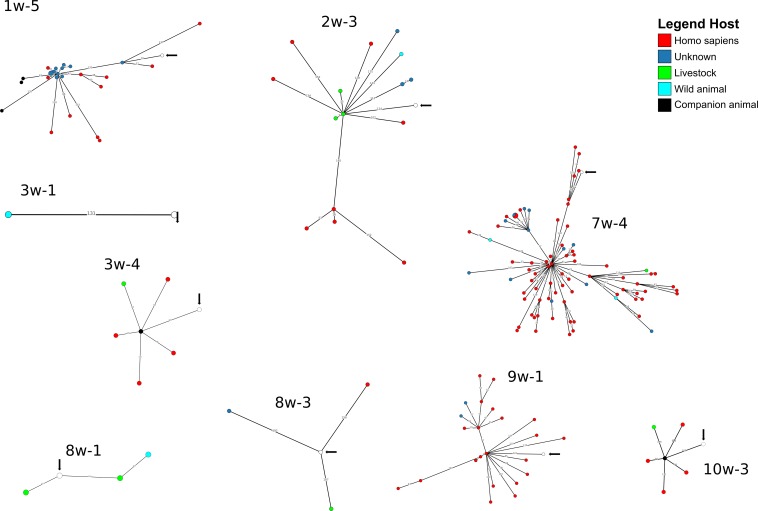
Depiction of cgMLST-based search for closest relatives of sequenced *E. coli* isolates. The search for closest relatives was performed using BacWGSTdb (Ruan and Feng, 2016). Different hosts are marked with different colors of the isolates. The water isolate from this study is indicated with a white circle and an arrow.

A cgMLST-based analysis was performed for *A. baumannii, E. coli* and *K. pneumoniae* isolates (*n* = 27) using BacWGSTdb. For 12/27 isolates (*E. coli n* = 9, *K. pneumoniae, n* = 2; *A. baumannii, n* = 1), close relatives were detected within the threshold of 200 cgMLST allele differences (Figures 3, 4 and Supplementary Table S8). Of the *E. coli* isolates, 6/9 were closely related to animal isolates, while two isolates were related to *E. coli* from human sources. The *A. baumannii* isolate was closely related to an isolate from a human sample, while both *K. pneumoniae* isolates exhibited a high similarity to isolates of human origin. These results are concordant with data from other European countries that have shown similarity of human isolates with water isolates (Jorgensen et al., 2017).

Our study should raise the awareness of the public and the regulatory bodies on the problem of the presence of clinically relevant antibiotic-resistant bacteria in water samples and their impact on human health. Indeed, an associated documentary based on this data (Baars and Lambrecht, 2018) has raised social and political discussions regarding the possible role of water for the dissemination of multidrug resistance in a One health context, and its implication on human health.

The aim of this study was to examine the presence of ESBL/carbapenemase-producing isolates in different water bodies, including recreational water, and to compare their genetic content and relationship to isolates from humans and animals. We were not only able to show that ESBL-, carbapenemase- and mobile colistin resistance gene-encoding isolates are present in water samples, but also that they harbored clinically relevant species displaying high rates of multidrug-resistance. These MDR bacteria were frequently associated with human disease, as for e.g., a hypervirulent *K. pneumoniae* ST268.

These findings suggest that there is already presence and dispersion of clonal isolates of clinically relevant bacteria in surface waters. In addition, the presence of isolates/antibiotic resistance genes of human and animal origin in water bodies (e.g., *bla*_VIM–__1_, human; *mcr-1*, animal) suggests that water itself may be regarded as a reservoir for the transmission and the exchange of isolates and antibiotic resistance genes originating from both human clinical sources and animal husbandry.

The prevalence and the real impact of MDR clinically relevant isolates derived from water bodies on human health is hard to estimate. However, there are reports on outbreaks caused by Carbapenemase-producing Enterobacterales/Enterobacteriaceae deriving from water samples (Carstens et al., 2014) as well as highly-related isolates from water sources and patient samples (Lepuschitz et al., 2019). Thus, there exists a risk of being colonized or, depending on the individual health status and the intensity of exposure, even infected through contact with contaminated water.

Our study has some limitations. Firstly, only ESBL/Carbapenemase-producers in the water samples were determined but not the overall number of isolates. Thus, the prevalence of ESBL/Carbapenemase-producers cannot be determined. Secondly, the study was designed as an observational study; therefore, the number of sequenced isolates was too low to perform statistical analyses.

## Conclusion

As clinically relevant antibiotic-resistant Gram-negative isolates were detected in the water samples examined, it is important to increase our knowledge concerning water sources as reservoirs and disseminators of such isolates. Additional investigations to quantify the transmission between the different environmental compartments are now highly warranted.

## Data Availability Statement

The datasets generated and analyzed for this study can be found in the European Nucleotide Archive (ENA) under the project accession number PRJEB29745.

## Author Contributions

LF, CB, OL, TB, SH, TC, and CI designed the study. CB and OL collected the data and samples. SH and TB processed the samples. LF performed antibiotic resistance and species determination. LF, JF, TC, and CI performed WGS of the isolates. LF, OS, JS, CB, OL, JF, TC, and CI analyzed the data. LF, TC, and CI wrote the manuscript that was critically reviewed and approved by all authors.

## Conflict of Interest

The authors declare that this study received funding from Norddeutscher Rundfunk, Germany. The funder had the following involvement in the study: study design, data collection, and analysis. LF received lecture fees from VTA, Austria.

## Footnotes

1 https://bigsdb.pasteur.fr/cgi-bin/bigsdb/bigsdb.pl?db=pubmlst_klebsiella_seqdef_public

2 https://pubmlst.org/abaumannii/

3 https://enterobase.warwick.ac.uk/species/index/ecoli
