# Supplementary material for: Multidrug-Resistant and Clinically Relevant Gram-Negative Bacteria Are Present in German Surface Waters
Source: Front Microbiol. 2019 Nov 29;10:2779. doi: 10.3389/fmicb.2019.02779 (PMC6896662; doi:10.3389/fmicb.2019.02779)
Supplement: Supplementary file 1 [file Data_Sheet_1.pdf]

Supplementary Material

1 Supplementary Data

Not applicable

2 Supplementary Figures and Tables

Supplementary table 1: Description of sampling sites. All but number nine are displayed in Supplementary Figure 1.

#	Sampling date	Sampling site	Sampling site detail
1	10/10/2017	River after urban wastewater treatment plant	On the bank of the stream (mid-size river); on the side of the treatment plant, about 500 meters behind the treatment plant.
2	10/10/2017	Lake for recreational use	At a small beach (official bathing area).
3	10/10/2017	River at a plant for meat and bone meal	On the bank of a small river receiving the treated sewage; on the opposite side of the plant, about 50 meters behind the discharge of the plant.
4	10/10/2017	Brook at agriculturally used area (field)	In a brook directly on the edge of the field.
5	10/10/2017	Brook at agriculturally used area (field)	In a brook between a small housing estate and fields, about 50 meters downstream from the nearest field.
6	10/11/2017	Reservoir lake for recreational use	At a beach (official bathing area)
7	10/11/2017	River downstream of discharged wastewater	On the bank of the stream (mid-size river); on the opposite side of the treatment plant, about 20 meters behind the outflow of the treatment plant.
8	10/11/2017	Brook near a meat (poultry) processing plant	In a brook about 200 meters behind the outflow of the treatment plant of the factory.
9	10/12/2017	Rivulet at a nursing home	In a brook about 20 meters behind the outflow of the treatment of the nursing home.
10	10/12/2017	River at a slaughterhouse	On the bank of the stream (mid-size river); about 20 meters behind the outflow of the treatment plant of the factory.
11	11/14/2017	Effluent of a hospital	Underground sewer at a large hospital
12	11/14/2017	River downstream of discharge of treated municipal wastewater	In the river directly after the outflow (about 20 meters distance) of the large urban wastewater treatment plant receiving the wastewater from the whole city and the regional hospital (see sampling site 11).

Supplementary table 2: Reference isolates for determination of *Enterobacter* species.

Species	Strain	Accession number
Enterobacter asburiae	ATCC 35953	CP011863
Enterobacter bugandensis	EB-247	FYBI00000000
Enterobacter cancerogenus	ATCC 33241	NZ_FYBA00000000
Enterobacter chengduensis	WCHECl-C4	MTSO00000000
Enterobacter chuandaensis	090028T	QZCS00000000
Enterobacter cloacae	ATCC 13047	CP001918
Enterobacter hormaechei	ATCC 49162	MKEQ00000000
Enterobacter huaxiensis	090008T	QZCT00000000
Enterobacter kobei	DSM 13645	CP017181
Enterobacter ludwigii	EN-119	CP017279
Enterobacter mori	LMG 25706	AEXB00000000
Enterobacter roggenkampii	DSM 16690	NZ_CP017184
Enterobacter sichuanensis	WCHECL1597	POVL00000000
Enterobacter soli	ATCC BAA-2102	LXES00000000
Enterobacter timonensis	mt20	FCOP00000000
Enterobacter xiangfangensis	LMG27195	CP017183

Supplementary table 3: Colony forming units in water and sediment samples obtained on ESBL Chromagar plates. The number of bacterial colonies was calculated to colonies per 100mL water or 1g sediment samples. *in these sampling sites, no sediment sample was taken.

Sampling site	Sample	Concentration (CFU/100 mL or 1g sediment sample)
1	water	11.5
	sediment	0
2	water	2.7
	sediment	0
3	water	15.8
	sediment	0
4	water	0
	sediment	0
5	water	0
	sediment	0
6	water	6.6
	sediment	0.1
7	water	68.5
	sediment	0
8	water	46.3
	sediment	15.0
9	water	41.8
	sediment	0
10	water	44.0
	sediment	0
11*	water	8130
12*	water	3865

Supplementary Table 4: Antimicrobial resistance testing data values of the seven species depicted in figure 2; the first number in the name of the isolates depicts, from which sampling site they were isolates (as described in Supplementary Table 3); s, source soil, w, source water; AM, Ampicillin; CAZ, Ceftazidim; CIP, Ciprofloxacin; CPD, Cefpodoxim; CTX, Cefotaxim; CXM, Cefuroxim; ETP, Ertapenem; GM, Gentamicin; IPM, Imipenem; MEM, Meropenem; MXF, Moxifloxacin; SAM, Ampicillin/Sulbactam; SXT, Trimethoprim/Sulfamethoxazol; TE, Tetracyclin; TGC, Tigecycline; TZP, Piperacillin/Tazobactam; (-) the MIC of these antibiotics was not determined. Sequenced isolates are marked in yellow.

**Isolate**	**Species**	**AM**	**CAZ**	**CIP**	**CPD**	**CTX**	**CXM**	**GM**	**ETP**	**IPM**	**MEM**	**MXF**	**SAM**	**SXT**	**TE**	**TGC**	**TZP**
1w-1	*Escherichia coli*	>=32	<=1	>=4	>=8	>=64	>=64	<=1	<=0,5	<=0,25	<=0,25	>=8	16	>=320	<=1	<=0,5	<=4
1w-2	*Escherichia coli*	>=32	16	<=0,25	>=8	>=64	>=64	<=1	<=0,5	<=0,25	<=0,25	2	16	>=320	<=1	<=0,5	<=4
1w-3	*Escherichia coli*	>=32	16	0,5	>=8	>=64	>=64	<=1	<=0,5	<=0,25	<=0,25	2	16	>=320	>=16	<=0,5	<=4
1w-4	*Escherichia coli*	>=32	<=1	<=0,25	>=8	>=64	>=64	<=1	<=0,5	<=0,25	<=0,25	<=0,25	16	<=20	<=1	<=0,5	<=4
1w-5	*Escherichia coli*	>=32	<=1	>=4	>=8	16	>=64	<=1	<=0,5	<=0,25	<=0,25	>=8	16	<=20	<=1	<=0,5	<=4
1w-6	*Escherichia coli*	>=32	4	>=4	>=8	>=64	>=64	<=1	<=0,5	<=0,25	<=0,25	>=8	16	>=320	>=16	<=0,5	<=4
1w-7	*Escherichia coli*	>=32	>=64	0,5	>=8	>=64	>=64	<=1	<=0,5	<=0,25	<=0,25	2	>=32	>=320	>=16	<=0,5	<=4
1w-8	*Escherichia coli*	>=32	<=1	0,5	>=8	>=64	>=64	<=1	<=0,5	<=0,25	<=0,25	2	<=2	<=20	<=1	<=0,5	<=4
1w-9	*Klebsiella oxytoca*	>=32	<=1	<=0,25	2	<=1	>=64	<=1	<=0,5	<=0,25	<=0,25	<=0,25	>=32	<=20	<=1	<=0,5	>=128

Isolate	Species	AM	C AZ	CIP	CP D	CTX	CXM	GM	ETP	IPM	MEM	MXF	SAM	SXT	TE	TGC	TZP
1w-10	*Klebsiella pneumoniae*	>=32	>= 64	2	>=8	>=64	>=64	<=1	<=0,5	<=0,25	<=0,25	2	>=32	>=320	>=16	2	>=12 8
1w-11	*Citrobacter freundii*	>=32	>= 64	<=0,25	>=8	>=64	>=64	<=1	<=0,5	<=0,25	<=0,25	2	>=32	<=20	<=1	<=0,5	16
1w-12	*Acinetobacter baumannii*	(-)	4	<=0,25	(-)	16	(-)	<=1	4(-)	<=0,25	0,5	(-)	<=2	<=20	2	<=0,5	8
1w-13	*Acinetobacter baumannii*	(-)	4	<=0,25	(-)	16	(-)	<=1	(-)4	<=0,25	<=0,25	(-)	<=2	<=20	<=1	<=0,5	<=4
1w-14	*Acinetobacter baumannii*	(-)	4	<=0,25	(-)	8	(-)	<=1	(-)4	<=0,25	<=0,25	(-)	<=2	<=20	<=1	<=0,5	<=4
1w-15	*Acinetobacter baumannii*	(-)	4	<=0,25	(-)	16	(-)	<=1	(-)4	<=0,25	<=0,25	(-)	<=2	<=20	<=1	<=0,5	8
1w-16	*Acinetobacter baumannii*	(-)	4	<=0,25	(-)	16	(-)	<=1	(-)4	<=0,25	<=0,25	(-)	<=2	<=20	<=1	<=0,5	8
1w-17	*Acinetobacter baumannii*	(-)	4	<=0,25	(-)	8	(-)	<=1	(-)4	<=0,25	<=0,25	(-)	<=2	<=20	<=1	<=0,5	8
1w-18	*Acinetobacter baumannii*	(-)	4	<=0,25	(-)	8	(-)	<=1	(-)4	<=0,25	<=0,25	(-)	<=2	<=20	<=1	<=0,5	<=4
1w-19	*Acinetobacter baumannii*	(-)	8	<=0,25	(-)	32	(-)	<=1	4(-)	<=0,25	<=0,25	(-)	<=2	<=20	<=1	<=0,5	8
2w-1	*Escherichia coli*	>=32	<= 1	0,5	>=8	>=64	>=64	<=1	<=0,5	<=0,25	<=0,25	2	16	<=20	<=1	<=0,5	<=4
2w-2	*Escherichia coli*	>=32	8	<=0,25	>=8	>=64	>=64	<=1	<=0,5	<=0,25	<=0,25	<=0,2 5	16	<=20	>=16	<=0,5	<=4
2w-3	*Escherichia coli*	>=32	<= 1	>=4	>=8	>=64	>=64	>=16	<=0,5	<=0,25	<=0,25	>=8	16	<=20	>=16	<=0,5	<=4
2w-4	*Acinetobacter baumannii*	(-)	4	<=0,25	(-)	8	(-)	<=1	4(-)	<=0,25	<=0,25	(-)	<=2	<=20	<=1	<=0,5	<=4

Isolate	Species	AM	CAZ	CIP	CPD	CTX	CXM	GM	ETP	IPM	MEM	MXF	SAM	SXT	TE	TGC	TZP
2w-5	*Acinetobacter baumannii*	(-)	4	<=0,25	(-)	16	(-)	<=1	(-)4	<=0,25	<=0,25	(-)	<=2	<=20	<=1	<=0,5	<=4
2w-6	*Acinetobacter baumannii*	(-)	4	<=0,25	(-)	16	(-)	<=1	(-)4	<=0,25	<=0,25	(-)	<=2	<=20	<=1	<=0,5	<=4
2w-7	*Acinetobacter baumannii*	(-)	4	<=0,25	(-)	8	(-)	<=1	(-)4	<=0,25	<=0,25	(-)	<=2	<=20	<=1	<=0,5	<=4
3w-1	*Escherichia coli*	>=32	4	<=0,25	>=8	>=64	>=64	<=1	<=0,5	<=0,25	<=0,25	<=0,25	>=32	>=320	<=1	<=0,5	<=4
3w-2	*Escherichia coli*	>=32	4	<=0,25	>=8	8	>=64	<=1	<=0,5	<=0,25	<=0,25	<=0,25	<=2	<=20	<=1	<=0,5	<=4
3w-3	*Escherichia coli*	>=32	<=1	<=0,25	>=8	4	>=64	<=1	<=0,5	<=0,25	<=0,25	<=0,25	16	<=20	<=1	<=0,5	<=4
3w-4	*Escherichia coli*	>=32	4	>=4	>=8	8	>=64	<=1	<=0,5	<=0,25	<=0,25	>=8	>=32	<=20	>=16	<=0,5	<=4
3w-5	*Escherichia coli*	>=32	<=1	>=4	>=8	8	>=64	<=1	<=0,5	<=0,25	<=0,25	>=8	16	>=320	>=16	<=0,5	<=4
3w-6	*Escherichia coli*	>=32	16	>=4	>=8	>=64	>=64	>=16	<=0,5	<=0,25	<=0,25	>=8	16	>=320	>=16	<=0,5	<=4
3w-7	*Escherichia coli*	>=32	4	>=4	>=8	>=64	>=64	<=1	<=0,5	<=0,25	<=0,25	>=8	<=2	<=20	<=1	<=0,5	<=4
3w-8	*Escherichia coli*	>=32	4	>=4	>=8	>=64	>=64	<=1	<=0,5	<=0,25	<=0,25	>=8	>=32	<=20	>=16	<=0,5	<=4
3w-9	*Klebsiella pneumoniae*	>=32	8	>=4	>=8	>=64	>=64	<=1	<=0,5	<=0,25	<=0,25	>=8	>=32	>=320	<=1	1	8
3w-10	*Klebsiella oxytoca*	>=32	<=1	>=4	4	<=1	>=64	<=1	<=0,5	<=0,25	<=0,25	>=8	>=32	<=20	<=1	<=0,5	>=128
3w-11	*Acinetobacter baumannii*	(-)	4	<=0,25	(-)	8	(-)	<=1	4(-)	<=0,25	<=0,25	(-)	<=2	<=20	<=1	<=0,5	<=4

Isolate	Species	AM	C<br>AZ	CIP	CP<br>D	CTX	CXM	GM	ETP	IPM	MEM	MXF	SAM	SXT	TE	TGC	TZP
3w-12	*Acinetobacter baumannii*	(-)	8	<=0,25	(-)	32	(-)	<=1	4(-)	<=0,25	0,5	(-)	<=2	<=20	2	<=0,5	16
3w-13	*Acinetobacter baumannii*	(-)	8	<=0,25	(-)	32	(-)	<=1	4(-)	<=0,25	0,5	(-)	<=2	<=20	2	<=0,5	32
3w-14	*Acinetobacter baumannii*	(-)	4	<=0,25	(-)	8	(-)	<=1	4(-)	<=0,25	<=0,25	(-)	<=2	<=20	<=1	<=0,5	<=4
3w-15	*Acinetobacter baumannii*	(-)	8	<=0,25	(-)	16	(-)	<=1	4(-)	<=0,25	<=0,25	(-)	<=2	<=20	<=1	<=0,5	8
3w-16	*Acinetobacter baumannii*	(-)	8	0,5	(-)	16	(-)	<=1	4(-)	<=0,25	<=0,25	(-)	<=2	<=20	<=1	<=0,5	16
3w-17	*Acinetobacter baumannii*	(-)	4	<=0,25	(-)	8	(-)	<=1	4(-)	<=0,25	<=0,25	(-)	<=2	<=20	<=1	<=0,5	<=4
3w-18	*Acinetobacter baumannii*	(-)	8	<=0,25	(-)	16	(-)	<=1	4(-)	<=0,25	<=0,25	(-)	<=2	<=20	<=1	<=0,5	<=4
6s-1	*Acinetobacter baumannii*	(-)	4	<=0,25	(-)	16	(-)	<=1	4(-)	<=0,25	<=0,25	(-)	<=2	<=20	<=1	<=0,5	<=4
6w-1	*Escherichia coli*	>=32	<=<br>1	<=0,25	>=8	>=64	>=64	<=1	<=0,5	<=0,25	<=0,25	<=0,2<br>5	16	>=320	>=16	<=0,5	<=4
6w-2	*Escherichia coli*	>=32	<=<br>1	<=0,25	>=8	8	>=64	<=1	<=0,5	<=0,25	<=0,25	<=0,2<br>5	16	>=320	<=1	<=0,5	<=4
6w-3	*Escherichia coli*	>=32	<=<br>1	<=0,25	>=8	>=64	>=64	<=1	<=0,5	<=0,25	<=0,25	<=0,2<br>5	16	>=320	<=1	<=0,5	<=4
6w-4	*Escherichia coli*	>=32	<=<br>1	<=0,25	>=8	16	>=64	<=1	<=0,5	<=0,25	<=0,25	<=0,2<br>5	16	<=20	<=1	<=0,5	<=4
6w-7	*Acinetobacter baumannii*	(-)	8	<=0,25	(-)	16	(-)	<=1	4	<=0,25	<=0,25	(-)	<=2	<=20	<=1	<=0,5	<=4
6w-8	*Acinetobacter baumannii*	(-)	>=<br>64	<=0,25	(-)	>=64	(-)	2	4	>=16	>=16	(-)	>=32	<=20	<=1	<=0,5	>=12<br>8

Isolate	Species	AM	CAZ	CIP	CPD	CTX	CXM	GM	ETP	IPM	MEM	MXF	SAM	SXT	TE	TGC	TZP
6w-9	*Escherichia coli*	(-)	16	<=0,25	(-)	16	(-)	<=1	(-)	<=0,25	<=0,25	(-)	<=2	<=20	<=1	<=0,5	<=4
6w-10	*Acinetobacter baumannii*	(-)	4	<=0,25	(-)	8	(-)	<=1	(-)4	<=0,25	<=0,25	(-)	<=2	<=20	<=1	<=0,5	<=4
7w-1	*Escherichia coli*	>=32	8	<=0,25	>=8	16	>=64	<=1	<=0,5 (-)	<=0,25	<=0,25	<=0,25	<=2	<=20	<=1	<=0,5	<=4
7w-2	*Escherichia coli*	16	4	<=0,25	>=8	8	>=64	<=1	4	<=0,25	<=0,25	<=0,25	<=2	<=20	<=1	<=0,5	<=4
7w-3	*Escherichia coli*	>=32	<=1	<=0,25	>=8	8	>=64	<=1	<=0,5	<=0,25	<=0,25	<=0,25	16	<=20	<=1	<=0,5	<=4
7w-4	*Escherichia coli*	>=32	4	>=4	>=8	>=64	>=64	<=1	<=0,5	<=0,25	<=0,25	>=8	<=2	<=20	<=1	<=0,5	<=4
7w-5	*Escherichia coli*	>=32	16	>=4	>=8	>=64	>=64	<=1	<=0,5	<=0,25	<=0,25	>=8	16	<=20	>=16	<=0,5	<=4
7w-6	*Escherichia coli*	>=32	16	>=4	>=8	>=64	>=64	<=1	<=0,5	<=0,25	<=0,25	>=8	16	<=20	>=16	<=0,5	<=4
7w-7	*Escherichia coli*	>=32	4	>=4	>=8	>=64	>=64	<=1	<=0,5	<=0,25	<=0,25	>=8	<=2	<=20	<=1	<=0,5	<=4
7w-8	*Escherichia coli*	>=32	8	<=0,25	>=8	>=64	>=64	<=1	<=0,5	<=0,25	<=0,25	<=0,25	8	>=320	>=16	<=0,5	<=4
7w-9	*Klebsiella pneumoniae*	>=32	<=1	0,5	>=8	>=64	>=64	>=16	<=0,5	<=0,25	<=0,25	0,5	16	>=320	<=1	<=0,5	<=4
7w-10	*Klebsiella pneumoniae*	>=32	<=1	<=0,25	>=8	>=64	>=64	<=1	<=0,5	<=0,25	<=0,25	<=0,25	16	<=20	<=1	<=0,5	<=4
7w-11	*Klebsiella pneumoniae*	>=32	16	<=0,25	>=8	>=64	>=64	<=1	<=0,5	<=0,25	<=0,25	<=0,25	16	>=320	<=1	1	<=4

Isolate	Species	AM	C AZ	CIP	CP D	CTX	CXM	GM	ETP	IPM	MEM	MXF	SAM	SXT	TE	TGC	TZP
7w-12	*Acinetobacter baumannii*	(-)	16	<=0,25	(-)	>=64	(-)	<=1	4(-)	<=0,25	<=0,25	(-)	16	>=320	8	>=8	8
7w-13	*Acinetobacter baumannii*	(-)	4	<=0,25	(-)	16	(-)	<=1	4(-)	<=0,25	<=0,25	(-)	<=2	<=20	<=1	<=0,5	<=4
7w-14	*Acinetobacter baumannii*	(-)	4	<=0,25	(-)	16	(-)	<=1	4(-)	<=0,25	<=0,25	(-)	<=2	<=20	<=1	<=0,5	<=4
7w-15	*Acinetobacter baumannii*	(-)	4	0,5	(-)	16	(-)	<=1	4(-)	<=0,25	0,5	(-)	<=2	<=20	<=1	<=0,5	8
7w-16	*Acinetobacter baumannii*	(-)	4	<=0,25	(-)	8	(-)	<=1	4(-)	<=0,25	<=0,25	(-)	<=2	<=20	<=1	<=0,5	<=4
7w-17	*Acinetobacter baumannii*	(-)	8	<=0,25	(-)	16	(-)	<=1	4(-)	<=0,25	<=0,25	(-)	<=2	<=20	<=1	<=0,5	8
7w-18	*Acinetobacter baumannii*	(-)	4	<=0,25	(-)	8	(-)	<=1	4(-)	<=0,25	<=0,25	(-)	<=2	<=20	<=1	<=0,5	<=4
7w-19	*Acinetobacter baumannii*	(-)	4	<=0,25	(-)	16	(-)	<=1	4(-)	<=0,25	<=0,25	(-)	<=2	<=20	<=1	<=0,5	8
8s-1	*Acinetobacter baumannii*	(-)	16	<=0,25	(-)	>=64	(-)	<=1	4(-)	<=0,25	<=0,25	(-)	16	>=320	8	>=8	8
8s-2	*Acinetobacter baumannii*	(-)	4	2	(-)	16	(-)	<=1	4(-)	<=0,25	<=0,25	(-)	<=2	<=20	>=16	4	<=4
8s-3	*Pseudomonas aeruginosa*	(-)	4	<=0,25	(-)	8	(-)	<=1	(-)	<=0,25	<=0,25	(-)	(-)	<=20	<=1	(-)	<=4
8s-4	*Acinetobacter baumannii*	(-)	2	<=0,25	(-)	>=64	(-)	<=1	4(-)	0,5	>=16	(-)	>=32	<=20	4	4	<=4
8s-5	*Acinetobacter baumannii*	(-)	4	<=0,25	(-)	16	(-)	<=1	4(-)	<=0,25	<=0,25	(-)	<=2	<=20	<=1	<=0,5	<=4
8s-6	*Acinetobacter baumannii*	(-)	4	<=0,25	(-)	8	(-)	<=1	4(-)	<=0,25	<=0,25	(-)	<=2	<=20	<=1	<=0,5	<=4
8s-7	*Acinetobacter baumannii*	(-)	4	<=0,25	(-)	8	(-)	<=1	4(-)	<=0,25	<=0,25	(-)	<=2	<=20	<=1	<=0,5	<=4

**Isolate**	**Species**	**AM**	**CAZ**	**CIP**	**CPD**	**CTX**	**CXM**	**GM**	**ETP**	**IPM**	**MEM**	**MXF**	**SAM**	**SXT**	**TE**	**TGC**	**TZP**
8s-8	*Acinetobacter baumannii*	(-)	4	<=0,25	(-)	16	(-)	<=1	4(-)	<=0,25	<=0,25	(-)	<=2	<=20	<=1	<=0,5	<=4
8w-1	*Escherichia coli*	>=32	16	0,5	>=8	>=64	>=64	<=1	>=8	<=0,25	1	<=0,25	<=2	<=20	>=16	4	8
8w-2	*Acinetobacter baumannii*	(-)	16	>=4	(-)	>=64	(-)	>=16	4	<=0,25	<=0,25	(-)	16	>=320	>=16	<=0,5	<=4
8w-3	*Escherichia coli*	>=32	<=1	>=4	>=8	8	>=64	>=16	<=0,5	<=0,25	<=0,25	>=8	16	>=320	>=16	<=0,5	<=4
8w-4	*Escherichia coli*	>=32	4	<=0,25	>=8	8	>=64	<=1	<=0,5	<=0,25	<=0,25	1	4	>=320	>=16	<=0,5	<=4
8w-5	*Escherichia coli*	>=32	4	>=4	>=8	>=64	>=64	<=1	<=0,5	<=0,25	<=0,25	>=8	16	<=20	>=16	<=0,5	<=4
8w-6	*Escherichia coli*	>=32	<=1	1	>=8	16	>=64	<=1	<=0,5	<=0,25	<=0,25	2	16	<=20	>=16	<=0,5	<=4
8w-8	*Escherichia coli*	>=32	4	>=4	>=8	32	>=64	<=1	<=0,5	<=0,25	<=0,25	>=8	<=2	<=20	<=1	<=0,5	<=4
8w-9	*Klebsiella pneumoniae*	>=32	4	1	>=8	4	16	>=16	<=0,5	<=0,25	<=0,25	2	16	>=320	>=16	1	<=4
8w-10	*Klebsiella pneumoniae*	>=32	4	<=0,25	>=8	>=64	>=64	<=1	<=0,5	<=0,25	<=0,25	0,5	>=32	<=20	<=1	<=0,5	<=4
8w-11	*Klebsiella pneumoniae*	>=32	4	1	>=8	4	16	>=16	<=0,5	<=0,25	<=0,25	2	16	>=320	>=16	1	16
8w-12	*Acinetobacter baumannii*	(-)	4	<=0,25	(-)	8	(-)	<=1	4(-)	<=0,25	<=0,25	(-)	<=2	<=20	<=1	<=0,5	<=4
8w-13	*Acinetobacter baumannii*	(-)	16	1	(-)	>=64	(-)	<=1	4(-)	<=0,25	0,5	(-)	<=2	>=320	>=16	<=0,5	8
8w-14	*Acinetobacter baumannii*	(-)	4	>=4	(-)	16	(-)	<=1	4(-)	<=0,25	<=0,25	(-)	<=2	<=20	>=16	2	8
8w-15	*Acinetobacter baumannii*	(-)	4	2	(-)	16	(-)	<=1	4(-)	<=0,25	<=0,25	(-)	<=2	<=20	>=16	4	<=4

Isolate	Species	AM	CAZ	CIP	CPD	CTX	CXM	GM	ETP	IPM	MEM	MXF	SAM	SXT	TE	TGC	TZP
8w-16	*Acinetobacter baumannii*	(-)	4	<=0,25	(-)	8	(-)	<=1	4(-)	<=0,25	<=0,25	(-)	<=2	<=20	<=1	<=0,5	<=4
8w-17	*Acinetobacter baumannii*	(-)	4	<=0,25	(-)	8	(-)	<=1	4(-)	<=0,25	<=0,25	(-)	<=2	<=20	<=1	<=0,5	<=4
8w-18	*Acinetobacter baumannii*	(-)	8	0,5	(-)	32	(-)	<=1	4(-)	<=0,25	0,5	(-)	<=2	<=20	8	1	64
8w-19	*Acinetobacter baumannii*	(-)	16	2	(-)	16	(-)	<=1	4(-)	<=0,25	0,5	(-)	<=2	<=20	2	2	<=4
9w-1	*Escherichia coli*	>=32	4	<=0,25	>=8	>=64	>=64	<=1	<=0,5(-)	<=0,25	<=0,25	0,5	<=2	<=20	<=1	<=0,5	<=4
9w-2	*Escherichia coli*	>=32	4	<=0,25	>=8	>=64	>=64	<=1	<=0,5 <=0,5	<=0,25	<=0,25	1	<=2	<=20	<=1	<=0,5	<=4
9w-3	*Escherichia coli*	>=32	<=1	<=0,25	>=8	>=64	>=64	<=1	<=0,5 <=0,5	<=0,25	<=0,25	<=0,25	>=32	<=20	>=16	<=0,5	<=4
9w-5	*Escherichia coli*	>=32	4	<=0,25	>=8	>=64	>=64	<=1	<=0,5 <=0,5	<=0,25	<=0,25	0,5	<=2	<=20	<=1	<=0,5	<=4
9w-6	*Escherichia coli*	>=32	4	<=0,25	>=8	>=64	>=64	<=1	<=0,5 <=0,5	<=0,25	<=0,25	1	<=2	<=20	<=1	<=0,5	<=4
9w-7	*Escherichia coli*	>=32	4	<=0,25	>=8	>=64	>=64	<=1	<=0,5 <=0,5	<=0,25	<=0,25	1	<=2	<=20	<=1	<=0,5	<=4
9w-9	*Pseudomonas aeruginosa*	(-)	8	>=4	(-)	>=64	(-)	8	(-)<=0,5	>=16	>=16	(-)	(-)	>=320	>=16	(-)	16
9w-13	*Escherichia coli*	>=32	<=1	<=0,25	>=8	>=64	>=64	<=1	<=0,5	<=0,25	<=0,25	<=0,25	16	<=20	<=1	<=0,5	<=4

Isolate	Species	AM	CAZ	CIP	CPD	CTX	CXM	GM	ETP	IPM	MEM	MXF	SAM	SXT	TE	TGC	TZP
9w-14	*Escherichia coli*	>=32	<=1	<=0,25	>=8	>=64	>=64	<=1	<=0,5	<=0,25	<=0,25	<=0,25	16	<=20	<=1	<=0,5	<=4
9w-16	*Escherichia coli*	>=32	<=1	<=0,25	>=8	>=64	>=64	<=1	<=0,5	<=0,25	<=0,25	<=0,25	16	<=20	<=1	<=0,5	<=4
10w-2	*Escherichia coli*	>=32	4	<=0,25	>=8	>=64	>=64	<=1	<=0,5 <=0,5	<=0,25	<=0,25	2	<=2	>=320	>=16	<=0,5	<=4
10w-3	*Escherichia coli*	>=32	4	>=4	>=8	>=64	>=64	<=1	<=0,5 <=0,5	<=0,25	<=0,25	>=8	16	<=20	>=16	<=0,5	8
10w-4	*Escherichia coli*	16	4	<=0,25	>=8	8	32	<=1	4<=0,5	<=0,25	<=0,25	<=0,25	<=2	<=20	<=1	<=0,5	<=4
10w-5	*Pseudomonas aeruginosa*	(-)	4	<=0,25	(-)	8	(-)	<=1	(-)<=0,5	2	8	(-)	(-)	<=20	4	(-)	8
10w-6	*Acinetobacter baumannii*	(-)	4	<=0,25	(-)	8	(-)	<=1	4<=0,5	<=0,25	<=0,25	(-)	<=2	<=20	<=1	<=0,5	<=4
10w-7	*Acinetobacter baumannii*	(-)	4	<=0,25	(-)	16	(-)	<=1	4(-)	<=0,25	<=0,25	(-)	<=2	<=20	<=1	<=0,5	<=4
10w-8	*Acinetobacter baumannii*	(-)	4	<=0,25	(-)	8	(-)	<=1	4<=0,5	<=0,25	<=0,25	(-)	<=2	<=20	<=1	<=0,5	<=4
10w-9	*Acinetobacter baumannii*	(-)	4	<=0,25	(-)	8	(-)	<=1	4<=0,5	<=0,25	<=0,25	(-)	<=2	<=20	<=1	<=0,5	<=4
11w-2	*Klebsiella oxytoca*	>=32	>=64	1	>=8	4	32	2	<=0,54	2	1	2	>=32	<=20	2	<=0,5	>=128
12w-1	*Escherichia coli*	>=32	<=1	>=4	>=8	8	>=64	8	<=0,5(-)	<=0,25	<=0,25	>=8	>=32	>=320	>=16	<=0,5	<=4
12w-10	*Klebsiella oxytoca*	>=32	4	>=4	>=8	2	>=64	8	<=0,5(-)	2	<=0,25	>=8	>=32	<=20	2	2	64

Isolate	Species	AM	CAZ	CIP	CPD	CTX	CXM	GM	ETP	IPM	MEM	MXF	SAM	SXT	TE	TGC	TZP
12w-12	*Klebsiella pneumoniae*	>=32	<=1	1	>=8	4	>=64	<=1	<=0,5(-)	<=0,25	<=0,25	2	>=32	<=20	<=1	1	16
12w-2	*Escherichia coli*	>=32	4	<=0,25	>=8	16	>=64	<=1	<=0,5(-)	<=0,25	<=0,25	<=0,25	>=32	>=320	>=16	<=0,5	<=4
12w-5	*Enterobacter cloacae complex*	>=32	>=64	1	>=8	>=64	>=64	4	2(-)	8	>=16	2	>=32	<=20	>=16	4	>=128

Supplementary Table 5: Depiction of the plasmid incompatibility groups in the Enterobacteriaceae isolates identified using PlasmidFinder (Carattoli et al., 2014)

Isolate	Species	Plasmid incompatibility groups
1w-4	*Escherichia coli*	IncFIB(Mar), IncFII(29), IncI1, IncFIB(AP001918), IncHI1B, Col(MP18), ColRNAI, Col156, Col(MG828)
1w-5	*Escherichia coli*	Col(BS512), Col(MGD2), ColRNAI, Col156, Col(MG828)
1w-9	*Klebsiella oxytoca*	IncFIB(K), IncFII(Y), ColRNAI
1w-10	*Klebsiella pneumoniae*	IncFIA(HI1), IncFIB(K), IncFII(K), Col(MGD2), ColRNAI
2w-3	*Escherichia coli*	IncFII, IncFIB(AP001918), IncB/O/K/Z, ColRNAI
3w-1	*Escherichia coli*	IncFIC(FII), IncFIA, IncI1, IncFIB(AP001918), IncFIB(pHCM2), ColRNAI
3w-10	*Klebsiella oxytoca*	IncR, ColRNAI
3w-4	*Escherichia coli*	IncI1, Col(BS512), ColRNAI, IncX4, Col(MG828)
3w-9	*Klebsiella pneumoniae*	IncFIB(K), IncFII(K), ColRNAI
6w-2	*Escherichia coli*	IncI1
7w-11	*Klebsiella pneumoniae*	IncFIB(K), IncA/C2
7w-2	*Escherichia coli*	IncI1, IncFIB(pHCM2), IncX1, Col8282
7w-4	*Escherichia coli*	IncFII(pRSB107), IncFIA, IncFIB(AP001918), Col8282, Col156, Col(MG828)
8w-1	*Escherichia coli*	IncI1, p0111, ColRNAI, Col156, ColpVC, Col(MG828)
8w-11	*Klebsiella pneumoniae*	IncFIA(HI1), IncR, Col156, ColRNAI

Isolate	Species	Plasmid incompatibility groups
8w-3	*Escherichia coli*	IncHI2, IncFII(pRSB107), IncI1, IncFIB(AP001918), IncHI2A, TrfA, IncQ1, Col(MG828), Col156, ColRNAI
9w-1	*Escherichia coli*	IncFII(29), IncFIB(AP001918), Col156, IncB/O/K/Z
10w-3	*Escherichia coli*	IncFII, IncFIB(AP001918), Col(BS512), IncX4, ColRNAI
12w-5	*Citrobacter kobei*	IncHI1B(CIT),IncHI1A(CIT),IncFII(K),ColRNAI,ColRNAI

Supplementary Table 6: Depiction of virulence determinants present in water isolates grouped by functions. Virulence determinants were identified using the VFDB database. *Acinetobacter baumannii*, *E. kobei*, *K. oxytoca* and *Pseudomonas resinovorans* isolates did not harbour any virulence determinants and are therefore not included in the table. Colour code: black: gene/s present, white: gene/s not present

		adherence						Antimicrobial activity	Antiphagocytosis	Biosurfactant	Invasion	Iron Aquisition, transport					motility	Protease			Quorum sensing	Secretion			Toxin				Pathotype
Name	Species	Afa/Dr family	Dr adhesins	ECP	F1C fimbriae	FdeC	Type 1 fimbriae	Pyocyanin	Alginate	Rhamnolipid	Ibes	Enterobactin	Pyochelin	Salmochelin	Shu	Yersiniabactin	Flagella Pseudomonas	Alkaline protease	LasA	LasB	las, rhl	TTSS	xcp secretion system	HSI-1	ExoA, ExoS, ExoT, ExoY	PLC	Sat	ShET2	
10w-3	*E. coli*			■		■						■		■															ExPEC
1w-4	*E. coli*					■						■			■														
1w-5	*E. coli*			■		■						■				■													ExPEC
2w-3	*E. coli*			■		■						■																	
3w-1	*E. coli*			■	■	■	■					■		■		■													UPEC
3w-4	*E. coli*			■		■						■																	
6w-2	*E. coli*						■				■																		NMEC
7w-2	*E. coli*			■		■	■					■																	
7w-4	*E. coli*			■		■	■					■				■											■		ExPEC

		adherence						Antimicrobial activity	Antiphagocytosis	Biosurfactant	Invasion	Iron Aquisition, transport					motility	Protease			Quorum sensing	Secretion			Toxin				Pathotype
Name	Species	Afa/Dr family	Dr adhesins	ECP	F1C fimbriae	FdeC	Type 1 fimbriae	Pyocyanin	Alginate	Rhamnolipid	Ibes	Enterobactin	Pyochelin	Salmochelin	Shu	Yersiniabactin	Flagella Pseudomonas	Alkaline protease	LasA	LasB	las, rhl	TTSS	xcp secretion system	HSI-1	ExoA, ExoS, ExoT, ExoY	PLC	Sat	ShET2	
8w-1	*E. coli*			■		■	■					■																	
8w-3	*E. coli*			■		■	■					■		■															ExPEC
9w-1	*E. coli*	■	■	■		■	■				■					■											■	■	DAEC/ExPEC/NMEC
1w-10	*K. pneumoniae*															■													
3w-9	*K. pneumoniae*																												
7w-11	*K. pneumoniae*													■		■													hypervirulent *Klebsiella pneumoniae*
8w-11	*K. pneumoniae*																												
9w-9	*P. aeruginosa*							■	■	■			■				■	■	■	■	■	■	■	■	■	■			

Supplementary Table 7: Source determination for *Acinetobacter* isolates, using the PubMLST database and the Pasteur MLST scheme (https://pubmlst.org/abaumannii/); * these sequence types were newly assigned in this study, therefore only one isolate of this ST is present in the PubMLST database.

Isolate	MLST	Number of isolates in PubMLST	Source			
			human	animal	environment	unknown
1w-12	203	4	3	-	-	1
2w-4	155	11	2	9	-	1
3w-14	1017	1	-	1	-	-
6s-1	1321*	1*	-	-	1	-
6w-10	1322*	1*	-	-	1	-
7w-12	647	1	-	-	1	-
8s-2, 8s-4	1112	1	-	-	-	1
8w-16	690	4	-	3	-	1
8w-19	1323*	1*	-	-	1	-
10w-8	1324*	1*	-	-	1	-

Supplementary Table 8: Source determination of sequenced isolates using BacWGSTdb.

Isolate	Species	Isolates related by cgMLST (range of cgMLST allele difference)		Source of related isolates		
			Source of closest relative	human	animal	unknown
1w-5	*Escherichia coli*	25 (79-187)	unknown	11	3	11
1w-10	*Klebsiella pneumoniae*	3 (66-175)	human	3	0	0
1w-12	*Acinetobacter baumannii*	1 (51)	human	1	0	0
2w-3	*Escherichia coli*	15 (119-194)	animal	8	4	3
3w-1	*Escherichia coli*	1 (131)	animal	0	1	0
3w-4	*Escherichia coli*	6 (148-194)	animal	4	2	0
3w-9	*Klebsiella pneumoniae*	222 (13-167)	human	164	5	55
7w-4	*Escherichia coli*	82 (63-200)	human	67	3	12
8w-1	*Escherichia coli*	3 (113-195)	animal	0	3	0
8w-3	*Escherichia coli*	3 Isolates cgMLST (107-192)	animal	1	1	1

9w-1	*Escherichia coli*	23 isolates cgMLST (97-188 cgMLST alleles different)	human	21	0	2
10w-3	*Escherichia coli*	6 isolates cgMLST (151-197 cgMLST allele difference)	animal	4	2	0

2.1 Supplementary Figures

Supplementary Figure 1. Depiction of the sampling sites. Water samples are marked in blue, sediment samples in red. Sampling site 9 is not shown due to protection of data privacy.

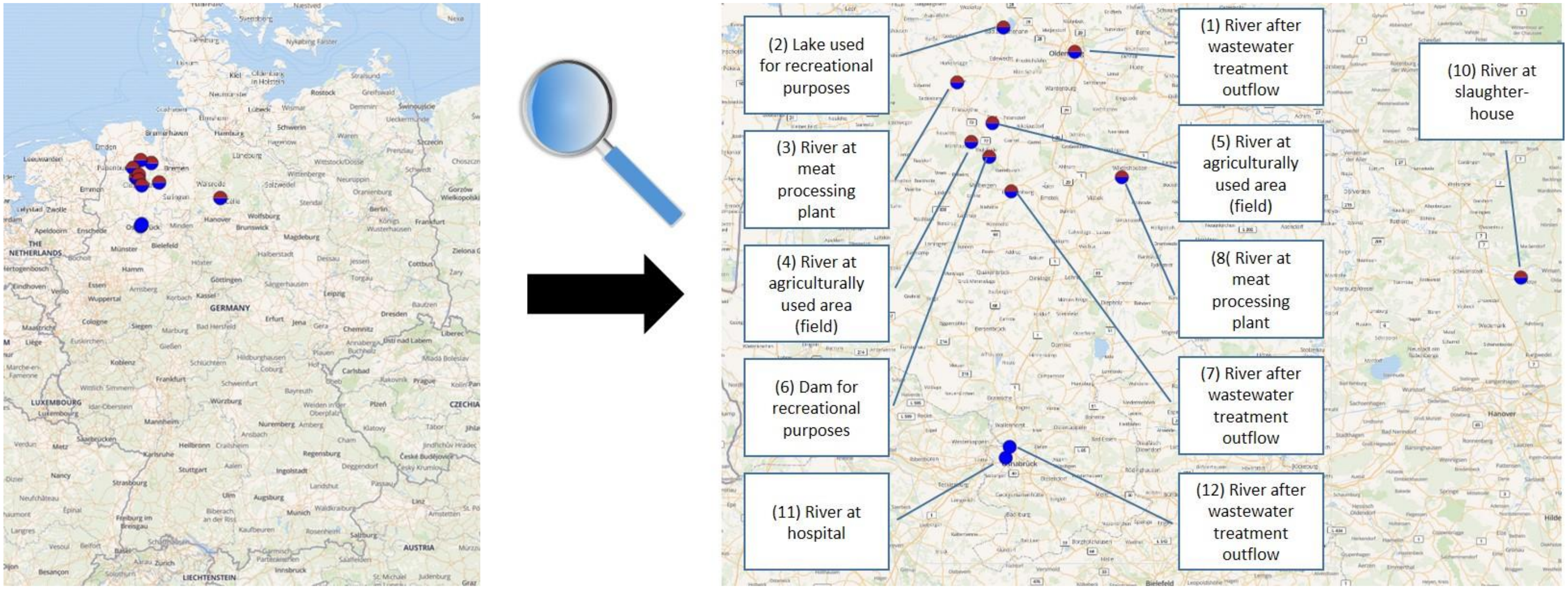

Supplementary Figure 2: Abundance of plasmid incompatibility groups among Enterobacterales. Plasmid incompatibility groups were determined using PlasmidFinder (Carattoli et al., 2014)

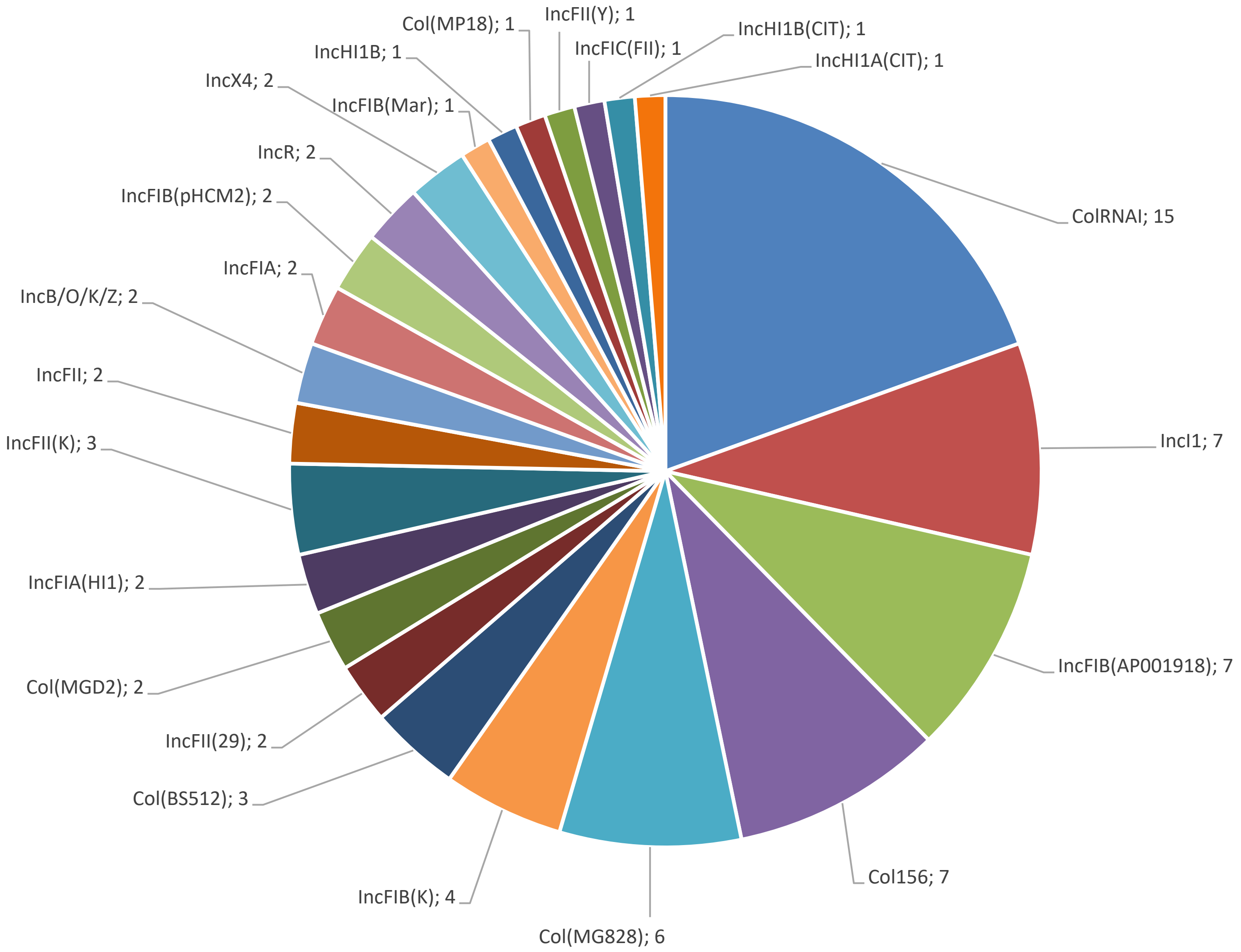

Supplementary Figure 3. Comparison of the *Klebsiella pneumoniae* 7w-11 genome with the hypervirulence plasmid pLVPK (A) and the ICE element (B) associated with hypervirulence in *Klebsiella pneumoniae* isolates.

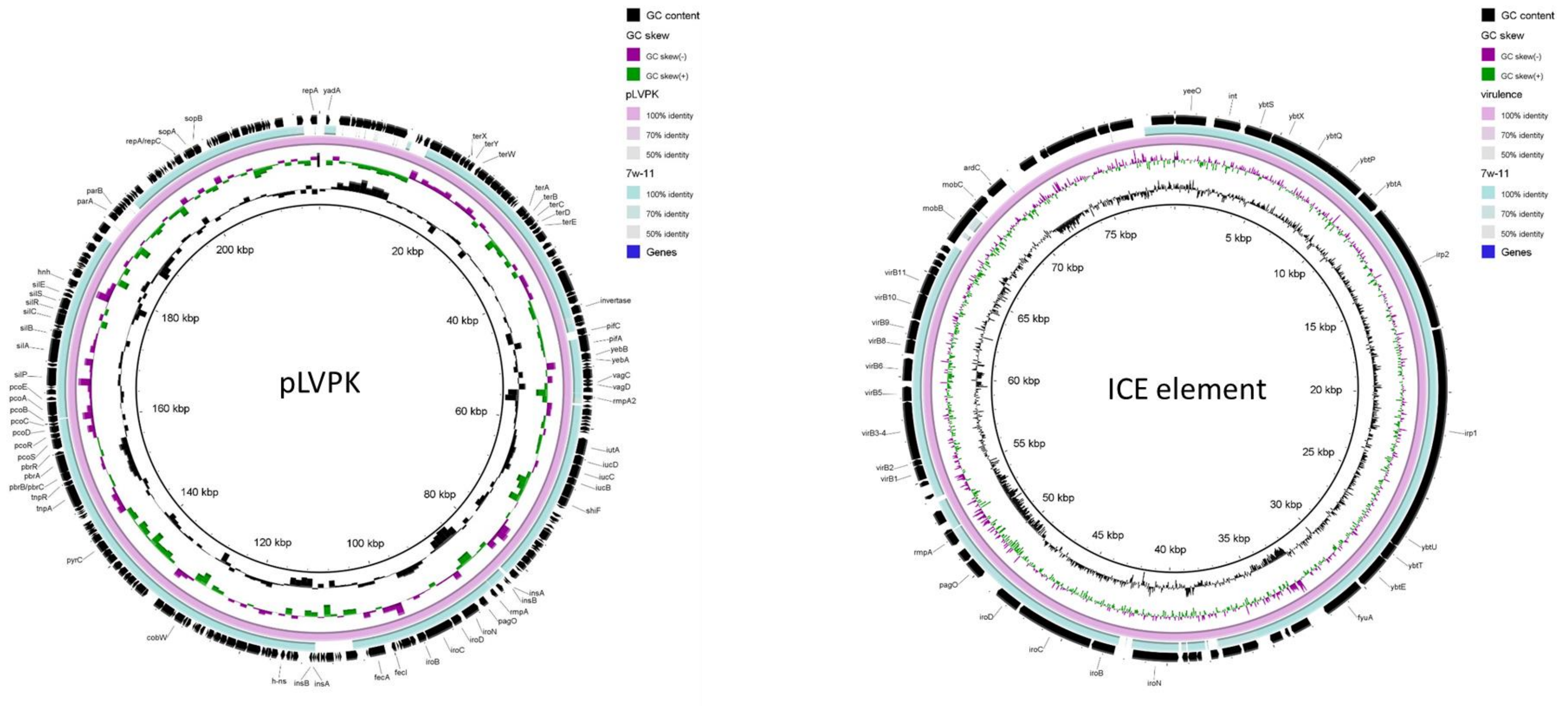

Supplementary References

Carattoli, A., Zankari, E., García-Fernández, A., Voldby Larsen, M., Lund, O., Villa, L., et al. (2014). In silico detection and typing of plasmids using PlasmidFinder and plasmid multilocus sequence typing. *Antimicrob. Agents Chemother.* 58, 3895–903. doi:10.1128/AAC.02412-14.
